# Threats to Validity in the Design and Conduct of Preclinical Efficacy Studies: A Systematic Review of Guidelines for In Vivo Animal Experiments

**DOI:** 10.1371/journal.pmed.1001489

**Published:** 2013-07-23

**Authors:** Valerie C. Henderson, Jonathan Kimmelman, Dean Fergusson, Jeremy M. Grimshaw, Dan G. Hackam

**Affiliations:** 1 Studies of Translation, Ethics and Medicine (STREAM) Group, Biomedical Ethics Unit, Department of Social Studies of Medicine, McGill University, Montréal, Québec, Canada; 2 Ottawa Hospital Research Institute, The Ottawa Hospital, Ottawa, Ontario, Canada; 3 Department of Medicine, University of Ottawa, Ottawa, Ontario, Canada; 4 Division of Clinical Pharmacology, Department of Medicine, University of Western Ontario, London, Ontario, Canada; Stanford University School of Medicine, United States of America

## Abstract

**Background:**

The vast majority of medical interventions introduced into clinical development prove unsafe or ineffective. One prominent explanation for the dismal success rate is flawed preclinical research. We conducted a systematic review of preclinical research guidelines and organized recommendations according to the type of validity threat (internal, construct, or external) or programmatic research activity they primarily address.

**Methods and Findings:**

We searched MEDLINE, Google Scholar, Google, and the EQUATOR Network website for all preclinical guideline documents published up to April 9, 2013 that addressed the design and conduct of in vivo animal experiments aimed at supporting clinical translation. To be eligible, documents had to provide guidance on the design or execution of preclinical animal experiments and represent the aggregated consensus of four or more investigators. Data from included guidelines were independently extracted by two individuals for discrete recommendations on the design and implementation of preclinical efficacy studies. These recommendations were then organized according to the type of validity threat they addressed. A total of 2,029 citations were identified through our search strategy. From these, we identified 26 guidelines that met our eligibility criteria—most of which were directed at neurological or cerebrovascular drug development. Together, these guidelines offered 55 different recommendations. Some of the most common recommendations included performance of a power calculation to determine sample size, randomized treatment allocation, and characterization of disease phenotype in the animal model prior to experimentation.

**Conclusions:**

By identifying the most recurrent recommendations among preclinical guidelines, we provide a starting point for developing preclinical guidelines in other disease domains. We also provide a basis for the study and evaluation of preclinical research practice.

*Please see later in the article for the Editors' Summary*

## Editors' Summary

**Background:**

The development process for new drugs is lengthy and complex. It begins in the laboratory, where scientists investigate the causes of diseases and identify potential new treatments. Next, promising interventions undergo preclinical research in cells and in animals (in vivo animal experiments) to test whether the intervention has the expected effect and to support the generalization (extension) of this treatment–effect relationship to patients. Drugs that pass these tests then enter clinical trials, where their safety and efficacy is tested in selected groups of patients under strictly controlled conditions. Finally, the government bodies responsible for drug approval review the results of the clinical trials, and successful drugs receive a marketing license, usually a decade or more after the initial laboratory work. Notably, only 11% of agents that enter clinical testing (investigational drugs) are ultimately licensed.

**Why Was This Study Done?:**

The frequent failure of investigational drugs during clinical translation is potentially harmful to trial participants. Moreover, the costs of these failures are passed onto healthcare systems in the form of higher drug prices. It would be good, therefore, to reduce the attrition rate of investigational drugs. One possible explanation for the dismal success rate of clinical translation is that preclinical research, the key resource for justifying clinical development, is flawed. To address this possibility, several groups of preclinical researchers have issued guidelines intended to improve the design and execution of in vivo animal studies. In this systematic review (a study that uses predefined criteria to identify all the research on a given topic), the authors identify the experimental practices that are commonly recommended in these guidelines and organize these recommendations according to the type of threat to validity (internal, construct, or external) that they address. Internal threats to validity are factors that confound reliable inferences about treatment–effect relationships in preclinical research. For example, experimenter expectation may bias outcome assessment. Construct threats to validity arise when researchers mischaracterize the relationship between an experimental system and the clinical disease it is intended to represent. For example, researchers may use an animal model for a complex multifaceted clinical disease that only includes one characteristic of the disease. External threats to validity are unseen factors that frustrate the transfer of treatment–effect relationships from animal models to patients.

**What Did the Researchers Do and Find?:**

The researchers identified 26 preclinical guidelines that met their predefined eligibility criteria. Twelve guidelines addressed preclinical research for neurological and cerebrovascular drug development; other disorders covered by guidelines included cardiac and circulatory disorders, sepsis, pain, and arthritis. Together, the guidelines offered 55 different recommendations for the design and execution of preclinical in vivo animal studies. Nineteen recommendations addressed threats to internal validity. The most commonly included recommendations of this type called for the use of power calculations to ensure that sample sizes are large enough to yield statistically meaningful results, random allocation of animals to treatment groups, and “blinding” of researchers who assess outcomes to treatment allocation. Among the 25 recommendations that addressed threats to construct validity, the most commonly included recommendations called for characterization of the properties of the animal model before experimentation and matching of the animal model to the human manifestation of the disease. Finally, six recommendations addressed threats to external validity. The most commonly included of these recommendations suggested that preclinical research should be replicated in different models of the same disease and in different species, and should also be replicated independently.

**What Do These Findings Mean?:**

This systematic review identifies a range of investigational recommendations that preclinical researchers believe address threats to the validity of preclinical efficacy studies. Many of these recommendations are not widely implemented in preclinical research at present. Whether the failure to implement them explains the frequent discordance between the results on drug safety and efficacy obtained in preclinical research and in clinical trials is currently unclear. These findings provide a starting point, however, for the improvement of existing preclinical research guidelines for specific diseases, and for the development of similar guidelines for other diseases. They also provide an evidence-based platform for the analysis of preclinical evidence and for the study and evaluation of preclinical research practice. These findings should, therefore, be considered by investigators, institutional review bodies, journals, and funding agents when designing, evaluating, and sponsoring translational research.

**Additional Information:**

Please access these websites via the online version of this summary at http://dx.doi.org/10.1371/journal.pmed.1001489.

- The US Food and Drug Administration provides information about drug approval in the US for consumers and for health professionals; its Patient Network provides a step-by-step description of the drug development process that includes information on preclinical research
- The UK Medicines and Healthcare Products Regulatory Agency (MHRA) provides information about all aspects of the scientific evaluation and approval of new medicines in the UK; its My Medicine: From Laboratory to Pharmacy Shelf web pages describe the drug development process from scientific discovery, through preclinical and clinical research, to licensing and ongoing monitoring
- The STREAM website provides ongoing information about policy, ethics, and practices used in clinical translation of new drugs
- The CAMARADES collaboration offers a “supporting framework for groups involved in the systematic review of animal studies” in stroke and other neurological diseases

## Introduction

The process of clinical translation is notoriously arduous and error-prone. By recent estimates, 11% of agents entering clinical testing are ultimately licensed [1], and only 5% of “high impact” basic science discoveries claiming clinical relevance are successfully translated into approved agents within a decade [2]. Such large-scale attrition of investigational drugs is potentially harmful to individuals in trials, and consumes scarce human and material resources [3]. Costs of failed translation are also propagated to healthcare systems in the form of higher drug costs.

Preclinical studies provide a key resource for justifying clinical development. They also enable a more meaningful interpretation of unsuccessful efforts during clinical development [4]. Various commentators have reported problems such as difficulty in replicating preclinical studies [5],[6], publication bias [7], and the prevalence of methodological practices that result in threats to validity [8].

To address these concerns, several groups have issued guidelines on the design and execution of in vivo animal experiments supporting clinical development (“preclinical efficacy studies”). Preclinical studies employ a vast repertoire of experimental, cognitive, and analytic practices to accomplish two generalized objectives [9]. First, they aim to demonstrate causal relationships between an investigational agent (treatment) and a disease-related phenotype or phenotype proxy (effect) in an animal model. Various factors can confound reliable inferences about such cause-and-effect relationships. For example, biased outcome assessment due to experimenter expectation can lead to spurious inferences about treatment response. Such biases present “threats to internal validity,” and are addressed by practices such as masking outcome assessors to treatment allocation.

The second aim of preclinical efficacy studies is to support generalization of treatment–effect relationships to human patients. This generalization can fail in two ways. Researchers might mischaracterize the relationship between experimental systems and the phenomena they are intended to represent. For instance, a researcher might err in using only rotational behavior in animals to represent human parkinsonism—a condition with a complex clinical presentation including tremor and cognitive symptoms. Such errors in theoretical relationships are “threats to construct validity.” Ways to address such threats include selecting well-justified model systems or outcome measures when designing preclinical studies, or confirming that the drug triggers molecular responses predicted by the theory of drug action.

Clinical generalization can also be threatened if causal mediators that are present in model systems are not present in patients. Responses in an inbred mouse, for example, may be particular to the strain, thus limiting generalizability to other mouse models or patients. Unforeseen factors that frustrate the transfer of cause-and-effect relationships from one system to another related system are “threats to external validity.” Researchers often address threats to external validity by replicating treatment effects in multiple model systems, or using multiple treatment formulations.

Many accounts of preclinical study design describe the concepts of internal and external validity. However, they often subsume the concept of “construct validity” under the label of “external validity.” We think that the separation of construct and external validity categories highlights the distinctiveness between the kinds of experimental operations that enhance clinical generalizability (see Box 1). Whereas addressing external validity threats involves conducting replication studies that vary experimental conditions, construct validity threats are reduced by articulating, addressing, and confirming theoretical presuppositions underlying clinical generalization.

**Box 1. Construct Validity and Preclinical Research**

Construct Validity concerns the degree to which inferences are warranted from the sampling particulars of an experiment (e.g., the units, settings, treatments, and outcomes) to the entities these samples are intended to represent. In preclinical research, “construct validity” has often been used to describe the relationship between behavioral outcomes in animal experiments and human behaviors they are intended to model (e.g., whether diminished performance of a rat in a “forced swim test” provides an adequate representation of the phenomenology of human depression).

Our analysis extends this more familiar notion to the animals themselves, as well as treatments and causal pathways. When researchers perform preclinical experiments, they are implicitly positing theoretical relationships between their experimental operations and the clinical scenario they are attempting to emulate. Clinical generalization is threatened whenever these theoretical relationships are in error.

There are several ways construct validity can be threatened in preclinical studies. First, preclinical researchers might use treatments, animal models, or outcome assessments that are poorly matched to the clinical setting, as when preclinical studies use an acute disease model to represent a chronic disease in human beings. Another way construct validity can be threatened is if preclinical researchers err in executing experimental operations. For example, researchers intending to represent intravenous drug administration can introduce a threat to construct validity if, when performing tail vein administration in rats, they inadvertently administer a drug subcutaneously. A third canonical threat to construct validity in preclinical research is when the physiological derangements driving human disease are not present in the animal models used to represent them. Note that, in all three instances, a preclinical study can—in principle—be externally valid if theories are adjusted. Studies in acute disease, while not “construct valid” for chronic disease, may retain generalizability for acute human disease.

To identify experimental practices that are commonly recommended by preclinical researchers for enhancing the validity of treatment effects and their clinical generalizations, we performed a systematic review of guidelines addressing the design and execution of preclinical efficacy studies. We then extracted specific recommendations from guidelines and organized them according to the principal type of validity threat they aim to address, and which component of the experiment they concerned. Based on the premise that recommendations recurring with the highest frequency represent priority validity threats across diverse drug development programs, we identified the most common recommendations associated with each of the three validity threat types. Additional aims of our systematic review are to provide a common framework for planning, evaluating, and coordinating preclinical studies and to identify possible gaps in formalized guidance.

## Methods

### Search Strategy

We developed a multifaceted search methodology to construct our sample of guidelines (See Table 1) from searches in MEDLINE, Google Scholar, Google, and the EQUATOR Network website. MEDLINE was searched using three strategies with unlimited date ranges up to April 2, 2013. Our first search (MEDLINE 1) used the terms “animals/and guidelines as topic.mp” and combined results with the exploded MeSH terms “research,” “drug evaluation, preclinical,” and “disease models, animal”. Our second search (MEDLINE 2) combined the results from four terms: “animal experimentation,” “models, animal,” “drug evaluation, preclinical,” and “translational research.” Results were limited to entries with the publication types “Consensus Development Conference,” “Consensus Development Conference, NIH,” “Government Publications,” or “Practice Guideline.” The third search (MEDLINE 3) combined the results of the exploded terms “animal experimentation,” “models, animal,” “drug evaluation, preclinical,” and “translational research” with the publication types “Consensus Development Conference,” “Consensus Development Conference, NIH,” and “Government Publications.”

**Table 1:** Summary of preclinical guidelines for in vivo experiments identified through various database searches.

Database Search or Source^a^	Date of Search/Acquisition	Unique Guidelines Identified^b^
MEDLINE 1	April 2, 2013	STAIR [10],[12] c
		Ludolph et al. [37]
		Rice et al. [38]
		Schwartz et al. [44]
		Verhagen et al. [45]
		García-Bonilla et al. [46]
		Kelloff et al. [47]
		Kamath et al. [48]
MEDLINE 2	April 2, 2013	Bellomo et al. [49]
MEDLINE 3	April 2, 2013	Moreno et al. [50]
Google Scholar	January 19, 2012	Scott et al. [25]
		Curtis et al. [51],[52] c
		Piper et al. [53]
		Liu et al. [54]
Google Scholar	April 9, 2013	Margulies and Hicks [36]
		Landis et al. [55]
Google	January 24, 2012	Bolon et al. [56]
		Macleod et al. [57]
		NINDS-NIH [58]
		Pullen et al. [59]
		Shineman et al. [60]
		Willmann et al. [40]
		Bolli et al. [61]
Correspondence	April 5–31, 2013	Grounds et al. [39]
		Savitz et al. [62],[63] c
		Katz et al. [64]

a No unique guidelines that had not been previously identified through previous search strategies were found by searching the EQUATOR Network or through hand searching of references in identified guidelines.

b The guidelines are listed under the search strategy by which they were first identified.

c Guidelines that were grouped together during analysis (e.g., identical guidelines that were published in more than one journal).

NINDS-NIH, US National Institutes of Health National Institute of Neurological Disorders and Stroke.

We conducted two Google Scholar searches. The first used the search terms “animal studies,” “valid,” “model,” and “guidelines” with no date restrictions. We limited our eligibility screening to the first 300 records, as returns became minimal after this point in screening. The second Google Scholar search was designed to identify preclinical efficacy guidelines that were published in the wake of the Stroke Therapy Academic Industry Roundtable (STAIR) guidelines—the best-known example of preclinical guidance. We searched for articles or statements citing the most recent STAIR guideline [10]. Results were screened for new guidelines. We also conducted a Google search seeking guidelines that might not be published in the peer-reviewed literature (e.g., granting agency statements). The terms “guidelines” and “preclinical” and “bias” were searched with no restrictions. We limited our eligibility screening to the first 400 records.

We searched the EQUATOR Network [11] website for guidelines, and reviewed the citations of included guidelines for additional guidelines. Authors of eligible guidelines were contacted for additional preclinical design/conduct guidelines.

### Eligibility Criteria

To be eligible, guidelines had to pertain to in vivo animal experiments. During title and abstract screening, we excluded guidelines that exclusively addressed (a) use of animals in teaching, (b) toxicology experiments, (c) testing of veterinary or agricultural interventions, (d) clinical experiments like assays on human tissue specimens, or (e) ethics or welfare, and guidelines that (f) did not offer targeted practice recommendations or (g) were strictly about reporting, rather than study design and conduct. We applied two further exclusion criteria during full-text screening. First, we excluded guidelines that did not address whole experiments, but merely focused on single elements of experiments (e.g., model selection): included guidelines must have recommended at least one practice aimed at addressing threats to internal validity (e.g., allocation concealment, selection of controls, or randomization). Second, we excluded guidelines listing four authors or fewer, except where articles reported using a formalized process to aggregate expert opinion (e.g., interviews). This was done to distinguish guidelines reflecting aggregated consensus from those reflecting the opinion of small teams of investigators. Where guidelines were later amended (e.g., [10],[12]) or where one guideline was published nearly verbatim in parallel venues (e.g., [13]–[15]), we consolidated the recommendations, and the group of related guidelines was treated as one unit during extraction and analysis. In the absence of well-characterized quality parameters for preclinical guideline documents (such as the AGREE II instrument for clinical guideline evaluation [16]), we did not include or exclude guidelines based on a quality score.

The application of our eligibility criteria was piloted in 100 citations to standardize implementation. Title and abstract screening of citations was conducted by one author (J. K. or V. C. H.). Guidelines meeting initial eligibility were screened by both J. K. and V. C. H. at the full-text level to ensure full eligibility for extraction.

### Extraction

We extracted discrete recommendations on the design and implementation of preclinical efficacy studies. These recommendations were categorized according to (a) which experimental component they concerned, using unit (animal), treatment, and outcome elements [17], and (b) the type of validity threat that they addressed, using the typology of validity described by Shadish et al. [9]. We also recorded the methodology used to develop the guidelines, and whether the guidelines cited evidence to support any recommendations.

Extraction was piloted by J. K., and each eligible guideline was extracted independently by two individuals (J. K. and V. C. H.). Extraction and categorization disagreements were resolved by discussion until consensus was reached.

In performing extractions, we made several simplifying assumptions. First, since nearly every recommendation has implications for all three validity types, we made inferences (when possible, based on explanations within the guidelines) about the type of validity threat authors seemed most concerned about when issuing a recommendation. Second, when guidelines offered nondescript recommendations to “blind experiments,” we assumed these recommendations pertained to blinded outcome assessment, not blinded treatment allocation. Third, some guidelines contained both reporting and design/conduct recommendations. We inferred that recommendations concerning reporting reflected tacit endorsements of certain design/conduct practices (i.e., the recommendation “report method of treatment allocation” was interpreted as suggesting that method of treatment allocation is relevant for inferential reliability, and, accordingly, randomized treatment allocation is to be preferred). Fourth, some recommendations could be categorized differently depending on whether an experiment was randomized or not. For example, the recommendation “characterize animals before study” (in relation to a variable disease status at baseline) addresses an internal validity threat for nonrandom studies, but a construct validity threat for studies using randomization, since variation would be randomly distributed across both arms. We assumed that such recommendations pertained to construct validity, since most preclinical efficacy studies are actively controlled, and many preclinical researchers intend phenotypes to be identical at baseline in treatment and control groups. Fifth, some guidelines explicitly endorsed another guideline in our sample. When this occurred, we assumed all recommendations in the endorsed previous guideline were recommended, regardless of whether the present guideline made explicit reference to the practices (see Table 2). Of our 26 included guidelines (see Table 1), 23 had contactable (i.e., not deceased, authorship reported) corresponding authors. We contacted authors to verify that we had comprehensively captured and accurately interpreted all recommendations contained in their guidelines; overall response rate of guideline authors was 58% (15/26).

**Table 2:** Results of recommendation extraction from guidelines addressing validity threats in preclinical experiments.

Recommendation Number	Validity Type	Application	Topic Addressed by the Recommendation	Number of Guidelines	General	Neurological and Cerebrovascular												Cardiac and Circulatory			Neuromuscular		Chemoprevention		Pain	Endometriosis	Arthritis	Sepsis	Renal Failure	Infectious Diseases
					Landis et al.	Ludolph et al.	NINDS-NIH	Scott et al.	Shineman et al.	Moreno et al.	Katz et al.	STAIR	Macleod et al.	Liu et al.^a^	García-Bonilla et al.	Savitz et al.	Margulies and Hicks^a^	Curtis et al.	Schwartz et al.	Bolli et al.	Willmann et al.	Grounds et al.	Verhagen et al.	Kelloff et al.	Rice et al.	Pullen et al.	Bolon et al.	Piper et al.	Bellomo et al.^b^	Kamath et al.
1	IV	U	Matching or balancing treatment allocation of animals	7				X	X	X	X										X							X	Δ	
2	IV	U	Standardized handling of animals	8	X			X	X												X	X			X	X	X			
3	IV	U	Randomized allocation of animals to treatment	20	X		X	X	X	X	X	X	X	Δ	X		Δ	X		X	X		X		X	X	X	X	Δ	
4	IV	U	Monitoring emergence of confounding characteristics in animals	12								X		Δ	X		Ŧ	X	X	X	X	X			X			X	Δ	
5	IV	U	Specification of unit of analysis	1	X																									
6	IV	T	Addressing confounds associated with anesthesia or analgesia	5										X	X			X							X		X			
7	IV	T	Selection of appropriate control groups	15	X		X		X	X					X	X		X	X			X	X		X		X	X	Δ	X
8	IV	T	Concealed allocation of treatment	14	X		X	X	X	X	X	X	X	Δ	X		Ŧ	X										X	Ŧ	
9	IV	T	Study of dose–response relationships	15		X	X		X	X	X	X		Ŧ		X	Δ	X	X				X	X		X	X			
10	IV	O	Use of multiple time points for measuring outcomes	5					X	X						X			X										X	
11	IV	O	Consistency of outcome measurement	8					X	X				X	X				X			X			X	X				
12	IV	O	Blinding of outcome assessment	20	X	X	X	X	X	X	X	X	X	Δ	X		Δ	X	X	X	X	X			X			X	Δ	
13	IV	Total	Establishment of primary and secondary end points	4	X				X		X												X							
14	IV	Total	Precision of effect size	6									X	X	X								X					X	Ŧ	
15	IV	Total	Management of interest conflicts	8		X	X		X		X	X	X	Δ			Ŧ													
16	IV	Total	Choice of statistical methods for inferential analysis	14	X	X	X	X	X	X	X		X	X	X			X	X	X			X							
17	IV	Total	Flow of animals through an experiment	16	X		X	X	X		X	X	X	Δ	X		Ŧ	X	X	X					X			X	Ŧ	
18	IV	Total	A priori statements of hypothesis	3					X									X					X							
19	IV	Total	Choice of sample size	23	X	X	X	X	X	X	X	X	X	Δ	X		Ŧ	X	X	X	X	X	X		X	X	X	X	Δ	
20	CV	U	Matching model to human manifestation of the disease	19		X		X	X	X	X	X		Δ	X	X	Ŧ	X	X	X					X	X	X	X	Δ	X
21	CV	U	Matching model to sex of patients in clinical setting	9						X	X	X		Ŧ	X	X	Δ	X							X					
22	CV	U	Matching model to co-interventions in clinical setting	7								X		Ŧ		X	Δ		X									X	Ŧ	
23	CV	U	Matching model to co-morbidities in clinical setting	10								X		Ŧ	X	X	Ŧ	X		X					X			X	Δ	
24	CV	U	Matching model to age of patients in clinical setting	11					X	X		X		Ŧ	X	X	Ŧ			X	X	X			X					
25	CV	U	Characterization of animal properties at baseline	20	X	X		X	X	X	X	X	X	Δ	X		Ŧ	X			X	X	X		X	X	X	X	Ŧ	
26	CV	U	Comparability of control group characteristics to those of previous studies	1		X																								
27	CV	T	Optimization of complex treatment parameters	5						X				X		X	X	X												
28	CV	T	Matching timing of treatment delivery to clinical setting	10			X		X		X	X		Ŧ		X	Δ			X								X	Ŧ	
29	CV	T	Matching route/method of treatment delivery to clinical setting	8		X	X					X		Ŧ		X	Ŧ		X				X							
30	CV	T	Pharmacokinetics to support treatment decisions	9		X			X		X	X		Ŧ		X	Δ		X						X					
31	CV	T	Matching the duration/exposure of treatment to clinical setting	10			X		X	X	X	X		Ŧ		X	Δ		X				X							
32	CV	T	Definition of treatment	2												X							X							
33	CV	T	Faithful delivery of intended treatment	6		X					X					X		X					X			X				
34	CV	T	Addressing confounds associated with treatment	9					X	X								X	X			X			X		X	X	Ŧ	
35	CV	O	Matching outcome measure to clinical setting	14		X					X	X		Ŧ		X	Ŧ		X	X	X		X		X		X	X	Δ	
36	CV	O	Degree of characterization and validity of outcome measure chosen	9		X			X	X	X			X	X			X				X	X							
37	CV	O	Treatment response along mechanistic pathway	15		X	X		X		X	X		Ŧ		X	Δ	X	X			X	X			X	X		X	
38	CV	O	Assessment of multiple manifestations of disease phenotype	10		X			X		X	X		Δ		X	Δ				X		X		X					
39	CV	O	Assessment of outcome at late/clinically relevant time points	7		X						X		Ŧ		X	Δ		X	X										
40	CV	O	Addressing treatment interactions with clinically relevant co-morbidities	1													X													
41	CV	O	Use of validated assay for molecular pathways assessment	1					X																					
42	CV	O	Definition of outcome measurement criteria	7		X									X			X	X		X		X				X			
43	CV	O	Addressing confounds associated with experimental setting	3	X					X												X								
44	CV	Total	Addressing confounds associated with setting	8					X	X					X			X			X	X			X		X			
45	EV	U	Replication in different models of the same disease	13		X			X		X	X		Δ		X	Δ	X		X			X	X				X	Ŧ	
46	EV	U	Replication in different species	8		X						X		Ŧ		X	Δ	X		X										X
47	EV	U	Replication at different ages	1													X													
48	EV	U	Replication at different levels of disease severity	1													X													
49	EV	T	Replication using variations in treatment	2														X	X											
50	EV	Total	Independent replication	12	X	X	X				X	X		Ŧ	X	X	Δ			X						X			X	
51	PROG	O	Inter-study standardization of end point choice	3				X										X			X									
52	PROG	Total	Define programmatic purpose of research	4		X			X												X							X		
53	PROG	Total	Inter-study standardization of experimental design	14		X				X	X			X	X	X	X	X		X	X	X				X	X			X
54	PROG	Total	Research within multicenter consortia	3							X						X			X										
55	PROG	Total	Critical appraisal of literature or systematic review during design phase	2		X																						X		

a Explicit endorsement of STAIR [10],[12].

b Explicit endorsement Piper et al. [53].

CV, threat to construct validity; EV, threat to external validity; IV, threat to internal validity; O, outcome; PROG, research program recommendations; T, treatment; Ŧ, recommendation imported from an endorsed guideline but not otherwise stated in the endorsing guideline; U, units (animals); Δ, recommendation imported from an endorsed guideline and also explicitly stated in the endorsing guideline; Total, all parts of the experiment; X, recommendation explicitly stated in the guideline.

NINDS-NIH, US National Institutes of Health National Institute of Neurological Disorders and Stroke.

### Data Synthesis

Discrete recommendations from each guideline were slotted into general recommendation categories. We confirmed that all extracted recommendations within a general category were consistent with one another. Recommendations were then reviewed by all study authors to determine whether some recommendations should be combined, and whether recommendations were categorized into appropriate validity types. All authors voted on each categorization; disagreements were resolved by discussion and consensus.

Data were synthesized by providing a matrix of the recommendations captured by each of the guidelines and were presented as simple presence or absence of the recommendation. The proportion of guidelines that addressed each recommendation was expressed as a simple proportion.

A PRISMA 2009 checklist for our review can be found in Checklist S1.

## Results

### Guideline Characteristics

**Figure 1:**
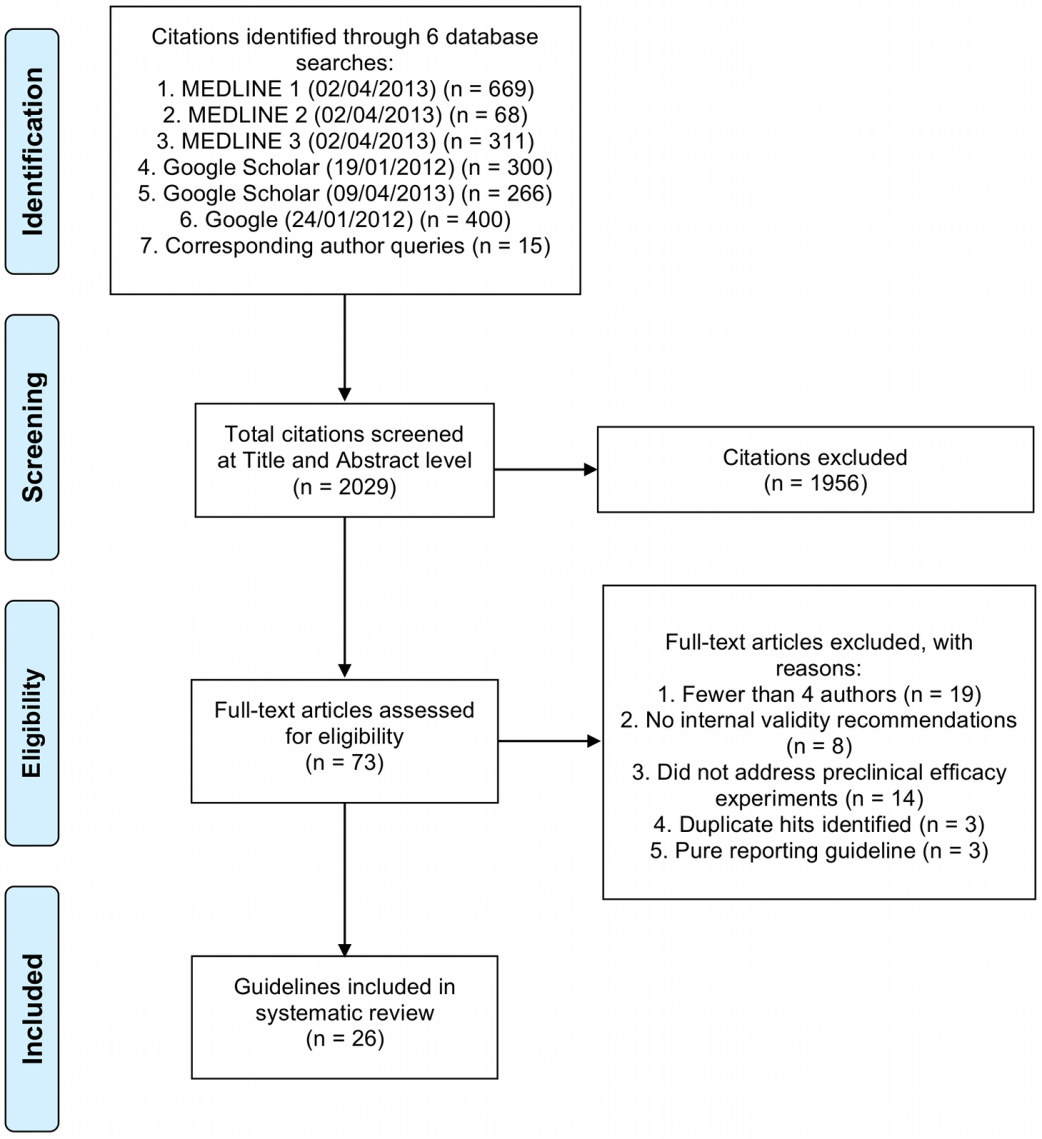
Flow of database searches and eligibility screening for guideline documents addressing preclinical efficacy experiments. Sample sizes at the identification stage reflect the raw output of the search and do not reflect the removal of duplicate entries between search strategies.

A total of 2,029 citations were identified by our literature search strategies. Of those, 73 met our initial screening criteria, and 26 guidelines on design of preclinical studies met our full eligibility criteria (see Figure 1). Almost all guidelines were published in the peer-reviewed literature (*n* = 25, 96%). In addition, we identified two guidelines [18],[19] addressing the synthesis of preclinical animal data (i.e., systematic review and meta-analysis). Given so few data, extraction and synthesis of these guidelines was not conducted.

Twelve guidelines on preclinical study design addressed various neurological and cerebrovascular drug development areas, and three addressed cardiac and circulatory disorders; other disorders covered in guidelines included sepsis, pain, and arthritis. Most guidelines (*n = *24, 92%) had been published within the last decade. Most were derived from workshop discussions, and only three described a clear methodology for their development. Though all but five guidelines (*n = *21, 81%) cited evidence in support of one or more recommendations, reference to published evidence supporting individual recommendations was sporadic.

Collectively, guidelines offered 55 different recommendations for preclinical design. On average, each guideline offered 18 recommendations (see Table 3). Fourteen recommendations were present in over 50% of relevant guidelines. The most common recommendations within each validity category are shown in Table 4. Recommendations contained in guidelines addressed all three components of preclinical efficacy studies—animals (units), treatments, and outcomes—though we counted more recommendations pertaining to the animals (148 in all) than to treatments (110) or outcomes (103). Many recommendations reflected in the 55 categories embodied a variety of particular experimental operations. In Table 4 we describe some of the many operations captured under a few representative recommendation categories.

**Table 3:** To what extent individual guidelines address each type of validity threat and make recommendations regarding the overall research program.

Category	Study	Number (Percent) of Recommendations Addressing Each Validity Type				Total (*n = *55)
		IV (*n = *19)	CV (*n = *25)	EV (*n = *6)	PROG (*n = *5)	
**General**	Landis et al.	10 (53)	2 (8)	1 (17)	0 (0)	13 (24)
**Neurological and cerebrovascular**	Ludolph et al.	5 (26)	12 (48)	3 (50)	3 (60)	23 (42)
	NINDS-NIH	9 (47)	4 (16)	1 (17)	0 (0)	14 (25)
	Scott et al.	8 (42)	2 (8)	0 (0)	1 (20)	11 (20)
	Shineman et al.	15 (79)	12 (48)	1 (17)	1 (20)	29 (53)
	Moreno et al.	10 (53)	10 (40)	0 (0)	1 (20)	21 (38)
	Katz et al.	10 (53)	11 (44)	2 (33)	2 (40)	25 (45)
	STAIR	8 (42)	14 (56)	3 (50)	0 (0)	25 (45)
	Macleod et al.	8 (42)	1 (4)	0 (0)	0 (0)	9 (16)
	Liu et al.	12 (63)	10 (40)	3 (50)	1 (20)	26 (47)
	García-Bonilla et al.	11 (58)	8 (32)	1 (17)	1 (20)	21 (38)
	Savitz et al.	3 (16)	16 (64)	3 (50)	1 (20)	23 (42)
	Margulies and Hicks	8 (42)	10 (40)	5 (83)	2 (40)	25 (45)
**Cardiac and circulatory**	Curtis et al.	11 (58)	11 (44)	3 (50)	2 (40)	27 (49)
	Schwartz et al.	9 (47)	10 (40)	1 (17)	0 (0)	20 (36)
	Bolli et al.	6 (32)	6 (24)	3 (50)	2 (40)	17 (31)
**Neuromuscular**	Willmann et al.	6 (32)	6 (24)	0 (0)	3 (60)	15 (27)
	Grounds et al.	6 (32)	7 (28)	0 (0)	1 (20)	14 (25)
**Chemoprevention**	Verhagen et al.	8 (42)	10 (40)	1 (17)	0 (0)	19 (35)
	Kelloff et al.	1 (5)	0 (0)	1 (17)	0 (0)	2 (4)
**Pain**	Rice et al.	9 (47)	10 (40)	0 (0)	0 (0)	19 (35)
**Endometriosis**	Pullen et al.	5 (26)	4 (16)	1 (17)	1 (20)	11 (20)
**Arthritis**	Bolon et al.	6 (32)	7 (28)	0 (0)	1 (20)	14 (25)
**Sepsis**	Piper et al.	9 (47)	7 (28)	1 (17)	2 (40)	19 (35)
**Renal failure**	Bellomo et al.	10 (53)	4 (16)	2 (33)	0 (0)	16 (29)
**Infectious diseases**	Kamath et al.	1 (5)	1 (4)	1 (17)	1 (20)	4 (7)

CV, threat to construct validity; EV, threat to external validity; IV, threat to internal validity; NINDS-NIH, US National Institutes of Health National Institute of Neurological Disorders and Stroke; PROG, research program recommendations.

**Table 4:** Most frequent recommendations appearing in preclinical research guidelines for in vivo animal experiments.

Validity Type	Recommendation Category	Examples	*n* (Percent) of Guidelines Citing
**Internal**	Choice of sample size	Power calculation, larger sample sizes	23 (89)
	Randomized allocation of animals to treatment	Various methods of randomization	20 (77)
	Blinding of outcome assessment	Blinded measurement or analysis	20 (77)
	Flow of animals through an experiment	Recording animals excluded from treatment through to analysis	16 (62)
	Selection of appropriate control groups	Using negative, positive, concurrent, or vehicle control groups	15 (58)
	Study of dose–response relationships	Testing above and below optimal therapeutic dose	15 (58)
**Construct**	Characterization of animal properties at baseline	Characterizing inclusion/exclusion criteria, disease severity, age, or sex	20 (77)
	Matching model to human manifestation of the disease	Matching mechanism, chronicity, or symptoms	19 (73)
	Treatment response along mechanistic pathway	Characterizing pathway in terms of molecular biology, histology, physiology, or behaviour	15 (58)
	Matching outcome measure to clinical setting	Using functional or non-surrogate outcome measures	14 (54)
	Matching model to age of patients in clinical setting	Using aged or juvenile animals	11 (42)
**External**	Replication in different models of the same disease	Different transgenics, strains, or lesion techniques	13 (50)
	Independent replication	Different investigators or research groups	12 (46)
	Replication in different species	Rodents and nonhuman primates	8 (31)
**Research Program** a	Inter-study standardization of experimental design	Coordination between independent research groups	14 (54)
	Defining programmatic purpose of research	Study purpose is preclinical, proof of concept, or exploratory	4 (15)

a Recommendations concerning the coordination of experimental design practices across a program of research.

### Threats to Internal Validity, Construct Validity, and External Validity

We identified 19 different recommendations addressing threats to internal validity, accounting for 35% of all 55 recommendations. The six most common are presented in Table 4. Practices endorsed in 50% or more guidelines but not reflected in Table 4 included the appropriate use of statistical methods and concealed allocation of treatment.

All guidelines, save one, contained recommendations to address construct validity threats. Twenty-five discrete construct validity recommendations were identified (Table 2), with the five most common presented in Table 4. Nine concerned matching the procedures used in preclinical studies—such as timing of drug delivery—to those planned for clinical studies. Three concerned directly addressing and ruling out factors that might impair clinical generalization, and another four involved confirming that experimental operations were implemented properly (e.g., if tail vein delivery of a drug is intended, confirming that the technically demanding procedure did not accidentally introduce the drug subcutaneously).

Recommendations concerning external validity threats were provided in 19 guidelines, and consisted of six recommendations. The most common was the recommendation that researchers reproduce their treatment effects in more than one animal model type, followed closely by independent replication of experiments (Table 4).

### Research Program Recommendations

Many guidelines contained recommendations that pertained to experimental *programs* rather than individual experiments. These programmatic or coordinating recommendations invariably implicated all three types of validity. In total, 17 guidelines (65%) contained at least one recommendation promoting coordinated research activities. For instance, 14 guidelines recommended the use of standardized experimental designs (54%), and two recommended critical appraisal (e.g., through systematic review) of prior evidence (8%). Such practices facilitate synthesis of evidence prior to clinical development, thereby enabling more accurate and precise estimates of treatment effect (internal validity), clarification of theory and clinical generalizability (construct validity), and exploration of causal robustness in humans (external validity).

## Discussion

We identified 26 guidelines that offered recommendations on the design and conduct of preclinical efficacy studies. Together, guidelines offered 55 prescriptions concerning threats to valid causal inference in preclinical efficacy studies. In recent years, numerous initiatives have sought to improve the reliability, interpretability, generalizability, and connectivity of laboratory investigations of new drugs. These include the establishment of preclinical data repositories [20], minimum reporting checklists for biomedical investigations [21], biomedical data ontologies [22], and reporting standards for animal studies [15]. Our review drew upon another set of initiatives—guidelines for the design and conduct of preclinical studies—to identify key experimental operations believed to address threats to clinical generalizability.

Numerous studies have documented that many of the recommendations identified in our study are not widely implemented in preclinical research. With respect to internal validity threats, a recent systematic analysis found that 13% and 14% of animals studies reported use of randomization or blinding respectively [23]. Several studies have revealed unaddressed construct validity threats in preclinical studies as well. For instance, one study found that the time between cardiac arrest and delivery of advanced cardiac life support is substantially shorter in preclinical studies than in clinical trials [24]. This represents a construct validity threat because the interval used in preclinical studies is not a faithful representation of that used in typical clinical studies. Similarly, most preclinical efficacy studies using the SOD1^G93A^ murine model for amyotrophic lateral sclerosis do not measure disease response directly, but instead measure random biologic variability, in part because of a lack of disease phenotype characterization (via quantitative genotyping of copy number) prior to the experiment [25].

The implementation of operations to address external validity has not been studied extensively. For instance, we are unaware of any attempts to measure the frequency with which preclinical studies used to support clinical translation are tested for their ability to withstand replication over variations in experimental conditions. Nevertheless, a recent commentary by a former Amgen scientist revealed striking problems with replication in preclinical experiments [5], and a systematic review of stroke preclinical studies found high variability in the number of experimental paradigms used to test drug candidates [26].

Whether failure to implement the procedures described above explains the frequent discordance between preclinical effect sizes and those in clinical trials is unclear. Certainly there is evidence that many practices captured in Table 2 are relevant in clinical trials [27],[28], and recommendations like those concerning justification of sample size or selection of models have an irrefutable logic. Several studies provide suggestive—if inconclusive—evidence that practices like unconcealed treatment allocation [29] and unmasked outcome assessment [30] may bias toward larger effect sizes in preclinical efficacy studies. Some studies have also investigated whether certain practices related to construct validity improve clinical predictivity. One study aggregated individual animal data from 15 studies of the stroke drug NXY-059 and found that when animals were hypertensive—a condition that is extremely common in acute stroke patients—effect sizes were greatly attenuated [31]. Another study suggested that nonpublication of negative studies resulted in an overestimation of effect sizes by one-third [7]. Though evidence that implementation of recommendations leads to better translational outcomes is very limited [32], we think there is a plausible case insofar as such practices have been shown to be relevant in the clinical realm [33].

We regard it as encouraging that distinct guidelines are available for different disease areas. Validity threats can be specific to disease domains, models, or intervention platforms. For instance, confounding of anesthetics with disease response presents a greater validity threat in cardiovascular preclinical studies than in cancer, since anesthetics can interact with cardiovascular function but rarely interfere with tumor growth. We therefore support customizing recommendations on preclinical research to disease domains or intervention platforms (e.g., cell therapy). By classing specific guideline recommendations into “higher order” experimental recommendations and identifying recommendations that are shared across many guidelines (see Table 4 and Checklist S2), our analysis provides researchers in other domains a starting point for developing their own guidelines. We further suggest that these consensus recommendations provide a template for developing consolidated minimal design/practice principles that would apply across all disease domains. Of course, developing such a guideline would require a formalized process that engages various preclinical research communities [21].

The practices identified above also provide a starting point for evaluating planned clinical investigations. In considering proposals to conduct early phase trials, ethics committees and investigators might use items identified in this report to evaluate the strength of preclinical evidence supporting clinical testing, or to prioritize agents for clinical development. We have created a checklist for the design and evaluation of preclinical studies intended to support clinical translation by identifying all design and research practices that are endorsed by guidelines in at least four different disease domains (Checklist S2). Funding agencies and ethics committees might use this checklist when evaluating applications proposing clinical translation. In addition, various commentators have called for a “science of drug development” [34]. Future investigations should determine whether the recommendations in our checklist and/or Table 4 result in treatment effect measurements that are more predictive of clinical response.

Our findings identify several gaps in preclinical guidance. We initially set out to capture guidelines addressing two levels of preclinical observation: individual experiments and aggregation of multiple experiments (i.e., systematic review of preclinical efficacy studies). However, because we were unable to identify a critical mass of guidelines addressing aggregation [18],[19], we could not advance these guidelines to extraction. The scarcity of this guidance type reveals a gap in the literature and could reflect the slow adoption of systematic review and meta-analytic procedures in preclinical research [35]. Second, guidelines are clustered in disease domains. For instance, just under half of the guidelines cover neurological or cerebrovascular diseases; none address cancer therapies—which have the highest rate of drug development attrition [1]. We think these gaps identify opportunities for improving the scientific justification of drug development: cancer researchers should consider developing guidelines for their disease domain, and researchers in all domains should consider developing guidelines for the synthesis of animal evidence. A third intriguing finding is the comparative abundance of recommendations addressing internal and construct validity as compared with recommendations addressing external validity. Where some guidelines urge numerous practices for addressing threats to external validity (e.g., guidelines for studies of traumatic brain injury [36], amyotrophic lateral sclerosis [37], and stroke [10],[12]), others offer none (e.g., guidelines for studies of pain [38] and Duchenne muscular dystrophy [39],[40]). As addressing external validity threats involves quasi-replication, guidelines could be more prescriptive regarding how researchers might better coordinate replication within research domains. Fourth, our findings suggest a need for formalizing the process of guideline development. In clinical medicine, there are elaborate protocols and processes for development of evidence-based guidelines [41],[42]. Very few of the guidelines in our sample used an explicit methodology, and use of evidence to support recommendations was sporadic.

Our analysis is subject to several important limitations. First, our search strategy may not have been optimal because of a lack of standardized terms for preclinical guidelines for in vivo animal experiments. We note that many eligible statements were not indexed as guidelines in databases, greatly complicating their retrieval. Both guideline authors and database curators should consider steps for improving the indexing of research guidelines. Second, experiments are systems of interlocking operations, and procedures directed at addressing one validity threat can amplify or dampen other validity threats. Dose–response curves, though aimed at supporting cause-and-effect relationships (internal validity), also clarify the mechanism of the treatment effect (construct validity) and define the dose envelope where treatment effects are reproducible (external validity). Our approach to classifying recommendations was based on what we viewed as the validity threat that guideline developers were most concerned about when issuing each recommendation, and our classification process was transparent and required the consensus of all authors. Further to this, slotting recommendations from guidelines into discrete categories of validity threat required a considerable amount of interpretation, and it is possible others would organize recommendations differently. Third, though many of the recommendations listed in Table 2 have counterparts in clinical research, it is important to recognize how their operationalization in preclinical research may be different. For instance, allocation concealment may necessitate steps in preclinical research that are not normally required in trials, such as masking various personnel involved in caring for the animals, delivering lesions or establishing eligibility, delivering treatment, and following animals after treatment. Last, our review excluded guidelines strictly concerned with reporting studies, and should therefore not be viewed as capturing all initiatives aimed at addressing the valid interpretation and application of preclinical research.

## Conclusions

We identified and organized consensus recommendations for preclinical efficacy studies using a typology of validity. Apart from findings mentioned above, the relationship between implementation of consensus practices and outcomes of clinical translation are not well understood. Nevertheless, by systematizing widely shared recommendations, we believe our analysis provides a more comprehensive, transparent, evidence-based, and theoretically informed rationale for analysis of preclinical studies. Investigators, institutional review boards, journals, and funding agencies should give these recommendations due consideration when designing, evaluating, and sponsoring translational investigations.

## Supporting Information

Checklist S1 **The PRISMA checklist.**
(DOC)

Checklist S2 **STREAM (Studies of Translation, Ethics and Medicine) checklist for design and evaluation of preclinical efficacy studies supporting clinical translation.**
(PDF)

## Note Added in Proof

It has come to our attention that the Nature Publishing Group has recently implemented reporting guidelines for new article submissions [43] that include a checklist to be completed by authors (http://www.nature.com/authors/policies/checklist.pdf).

## Abbreviations

STAIR: Stroke Therapy Academic Industry Roundtable
