# Supplementary material for: Threats to Validity in the Design and Conduct of Preclinical Efficacy Studies: A Systematic Review of Guidelines for In Vivo Animal Experiments
Source: PLoS Med. 2013 Jul 23;10(7):e1001489. doi: 10.1371/journal.pmed.1001489 (PMC3720257; doi:10.1371/journal.pmed.1001489)
Supplement: Checklist S2 — STREAM (Studies of Translation, Ethics and Medicine) checklist for design and evaluation of preclinical efficacy studies supporting clinical translation. (PDF) [file pmed.1001489.s002.pdf]

Validity Checklist for *in vivo* Efficacy Studies

Version 1.0

STREAM
Studies of Translation, Ethics and Medicine

This checklist concerns the design and conduct of *in vivo* preclinical studies that are performed to establish the clinical utility of new treatments ("efficacy studies"). In particular, the checklist is aimed at helping researchers, reviewers, and others determine whether a given experiment or a group of experiments has addressed factors that threaten the reliability and clinical generalizability of study findings. Items below should be clearly reported in publications. Note: This checklist has not undergone a formal development and validation process, including evaluations of face, criterion, and content validity.

Internal Validity

		Yes	No
**1a**	Were treatments allocated to animals using a randomized procedure?	☐	☐
	**1b** If "Yes", describe the method used to generate the randomization sequence:		
	**1c** If non-randomized allocation was used, were groups balanced by another characteristic (e.g. sex, age, disease status)?	☐	☐
**2**	Was the handling of animals before and during the experiment uniform (e.g. same handlers, standardized animal training)?	☐	☐
**3**	Was the emergence of confounding physiological variables monitored and addressed (e.g. blood pressure, body temperature)?	☐	☐
**4**	If anesthesia or analgesia were used during the experiment, were potential confounding effects on outcomes addressed?	☐	☐
**5**	Were appropriate controls used in all *in vivo* experiments (e.g. positive, negative)?	☐	☐
**6**	Was treatment allocation concealed from the investigators during the experiment?	☐	☐
**7**	Were investigators blinded to treatment allocation during outcome assessment?	☐	☐
**8**	Has a dose-response treatment effect been demonstrated?	☐	☐
**9**	Were measures taken to ensure that outcome assessment techniques were consistent and reproducible (e.g. training of research personnel, performing assessments in same location)?	☐	☐
**10**	Has precision of the treatment effect been justified, and is choice of measure of dispersion justified?	☐	☐
**11**	Are the statistical tests appropriate, reported in detail, and sufficiently justified?	☐	☐
**12**	Has every animal been accounted for, from entry into experiment through treatment, outcome assessment, sacrifice and analysis?	☐	☐
**13a**	Has a power calculation been performed *a priori* to determine sample size?	☐	☐
	**13b** If "No," how was the sample size chosen:		

External Validity

		Yes	No
**14**	Have treatment effects been demonstrated in more than one model?	☐	☐
**15**	Have treatment effects been replicated by an independent research group?	☐	☐

Construct Validity

		Yes	No
**16a**	Is the selected animal model the best available representation of human disease?	☐	☐
	**16b** If "Yes", on what basis?		
**17**	Did the experiment simulate the delivery of treatments that are co-administered in typical clinical settings?	☐	☐
**18**	Have comorbidities typical of clinical settings been simulated in the experiment?	☐	☐
**19**	Does the age of animals match the age of patients in relevant clinical settings?	☐	☐
**20a**	Were basic animal characteristics sufficiently defined at baseline (e.g. strain, sex, age, disease status)?	☐	☐
	**20b** Were predetermined inclusion and exclusion criteria used?	☐	☐
**21**	Has the timing of treatment administration been matched to the timing of administration anticipated in clinical settings?	☐	☐
**22**	Was appropriate delivery of treatment confirmed (e.g. to the appropriate organ system or compartment)?	☐	☐
**23**	Were confounders that might result from treatment addressed (e.g. effects of a drug on other systems, complications from treatment administration)?	☐	☐
**24**	Were the measures of disease response the best available representations of those used in clinical settings?	☐	☐
**25**	Have the outcome assessment techniques and criteria been validated and shown to be reproducible?	☐	☐
**26**	Have treatment effects been demonstrated using more than one measure of response?	☐	☐
**27**	Has treatment response been demonstrated at a mechanistic level?	☐	☐
**28**	Are there aspects of the experimental environment that could interfere with clinical generalization (e.g. stressors such as noise, housing)?	☐	☐

Research Program

		Yes	No
**29**	Was study design standardized such that results can be compared with similar preclinical studies?	☐	☐
